# Serum MMP‐3 and its association with central arterial stiffness among young adults is moderated by smoking and BMI

**DOI:** 10.14814/phy2.14920

**Published:** 2021-06-10

**Authors:** Nathaniel J. Iannarelli, Adam J. MacNeil, Kylie S. Dempster, Terrance J. Wade, Deborah D. O’Leary

**Affiliations:** ^1^ Department of Health Sciences Faculty of Applied Health Sciences Brock University Saint Catharines ON Canada; ^2^ Brock‐Niagara Centre for Health and Well‐Being Brock University Saint Catharines ON Canada

**Keywords:** arterial stiffness, body mass index, central arterial stiffness, matrix metalloproteinases, smoking

## Abstract

Central arterial stiffness is an independent predictor of cardiovascular disease. It is characterized by a marked reduction in the elastin‐collagen ratio of the arterial wall extracellular matrix (ECM), and is largely the result of degradation of various ECM components. Matrix metalloproteinase‐3 (MMP‐3) may contribute to central arterial stiffness via its involvement in ECM homeostasis and remodeling. This study examined the association between serum MMP‐3 concentrations and central arterial stiffness and potential interactions of MMP‐3 and traditional cardiovascular risk factors in a population of healthy young adults. A total of 206 participants (*n* = 109 females) aged 19–25 years were included in the current study. Central arterial stiffness was measured non‐invasively as carotid‐femoral pulse wave velocity (cfPWV) (m/s). MMP‐3 concentrations (ng/ml) were measured using ELISA techniques. Regression analyses were used to examine the association between cfPWV and MMP‐3, adjusting for age, sex, smoking status, body mass index (BMI), instantaneous mean arterial pressure (MAP) and heart rate, and serum C‐reactive protein. Interactions between MMP‐3 with smoking, BMI, sex, and MAP were analyzed in subsequent regression models. MMP‐3 was an independent predictor of cfPWV (*β* = 0.187, *p* = 0.007), and significant interactions between MMP‐3 and regular smoking (*β* = 0.291, *p* = 0.022), and MMP‐3 and BMI (*β* = 0.210, *p* = 0.013) were observed. Higher serum MMP‐3 concentrations were associated with a faster cfPWV and thus, greater central arterial stiffness. Interactions between MMP‐3 and smoking, and MMP‐3 and BMI may, in part, drive the association between MMP‐3 and central arterial stiffness.

## Graphical abstract

Higher concentrations of serum MMP‐3 are associated with a greater degree of central arterial stiffness in healthy young adults. This association is modulated by smoking and BMI, such that the association between serum MMP‐3 concentrations and cfPWV is greater in those who smoke regularly and those who have a greater BMI.

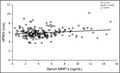

## 1 INTRODUCTION

Although originally considered as passive conduits for blood flow, the large elastic arteries (i.e., the aorta and its major branches) are now recognized as playing a critical role in the buffering of cyclic changes in blood pressure (BP) (Nichols & O’Rourke, 2005). Stiffening of these arteries, particularly the aorta, results in a number of adverse hemodynamic changes, including a widening of the pulse pressure (PP) and an increase in pulse wave velocity (PWV), which, in turn, promote vascular and cardiac remodeling and ultimately increase the risk of cardiovascular disease (CVD) (Nichols & O’Rourke, 2005). It has been shown that central arterial stiffness, as measured by carotid‐femoral pulse wave velocity (cfPWV), is an independent predictor of both CVD morbidity and all‐cause mortality across several populations, and as such, it is widely considered the “gold standard” measure of arterial stiffness (Laurent et al., 2006; Van Bortel et al., 2012). Several mechanisms have been implicated in the progression of central arterial stiffness; however, the exact mechanisms have not yet been fully elucidated.

Under normal physiological conditions, the large elastic arteries function to dampen pulsatile ventricular ejections into a near steady blood flow to the periphery, which serves to maintain adequate tissue perfusion, reduce left ventricular afterload, and improve coronary perfusion (Nichols & O’Rourke, 2005). This buffering function is largely dependent on the relative contributions of elastin and collagen (i.e., elastin‐collagen ratio) within the aortic wall extracellular matrix (ECM), in which elastin provides the main elastic element (Zieman et al., 2005). Normally, the relative quantities of these proteins are held within a homeostatic range by simultaneous processes of synthesis and degradation; however, dysregulation of this balance can lead to a marked reduction in the elastin‐collagen ratio and ultimately central arterial stiffness (Lyle & Raaz, 2017; Zieman et al., 2005). Several enzymes, including serine proteases and members of the matrix metalloproteinase (MMP) family, have been implicated in the regulation of the ECM by degrading its various constituents and thus, may play a functional role in the progression of arterial stiffness.

MMPs are a family of calcium‐ and zinc‐dependent endopeptidases that includes various collagenases, gelatinases, stromelysins, matrilysins, and membrane‐type MMPs (Raffetto & Khalil, 2008; Verma & Hansch, 2007). These enzymes are expressed by several cell types, including vascular endothelial and smooth muscle cells (VECs and VSMCs, respectively), fibroblasts, and various immune cells, including macrophages and T‐lymphocytes (Verma & Hansch, 2007). Collectively, MMPs are capable of degrading all components of the ECM and basement membrane and thus, play a critical role in tissue maintenance and remodeling (Chen et al., 2013). MMPs also regulate the secretion and activation of several chemokines, cytokines, and growth factors thereby playing a role in several physiological processes, such as fibrosis, inflammation, cellular apoptosis, and arterial wall remodeling (Loffek et al., 2011; Rajzer et al., 2017). MMP activity is regulated at the level of transcription, activation of precursor zymogens, and interactions with specific ECM components (Chen et al., 2013). Tissue inhibitors of MMPs (TIMPs) provide a particularly potent regulatory mechanism by which excessive ECM degradation is avoided (Verma & Hansch, 2007). Imbalance of the MMP‐TIMP system due to, for example, overexpression of MMPs can lead to pathological changes within the arterial wall ECM that are associated with arterial stiffness (Chen et al., 2013; Raffetto & Khalil, 2008).

MMP‐3 (stromelysin‐1) may be particularly important in the remodeling of the aorta due to its broad substrate spectrum, which includes most of the major constituents of the arterial wall, including elastin, types‐II, ‐IV, ‐V, ‐IX, and ‐X collagen, gelatin, laminin, fibronectin, and various proteoglycans (Nagase, 2013). MMP‐3 is also capable of activating various other MMPs (e.g., MMP‐1 and MMP‐9) (Medley et al., 2003), which may further exacerbate vascular remodeling and arterial stiffening. Thus, MMP‐3 may play a critical role in the progression of arterial stiffness through its direct effects on various ECM constituents as well as its indirect effects on the activation of downstream MMPs.

Evidence of the importance of MMP‐3 in central arterial stiffness was first demonstrated in a study examining the effects of the *MMP3* genotype determined by a 5A/6A promoter polymorphism on central arterial stiffness (Medley et al., 2003). This study found that homozygosity for either the 5A‐ or 6A‐allele was associated with a greater degree of central arterial stiffness and a higher systolic BP (SBP) in older (>60 years) adults (Medley et al., 2003). In terms of circulating MMP‐3, higher plasma concentrations of MMP‐3 have been associated with a faster cfPWV in hypertensive individuals (Rajzer et al., 2017) and higher serum concentrations of MMP‐3 have been associated with a faster cfPWV in type 1 diabetics (Peeters et al., 2017). To date, no studies have examined the association between central arterial stiffness and serum MMP‐3 in a healthy population. Accordingly, the purpose of the current study was to examine the association between serum MMP‐3 concentrations and cfPWV in a population of healthy young adults. It was hypothesized that higher serum MMP‐3 concentrations would be associated with a faster cfPWV and thus, a greater degree of central arterial stiffness.

## 2 MATERIALS AND METHODS

### 2.1 Study population and design

The current study was carried out as part of the ongoing Niagara Longitudinal Heart Study (NLHS), which is taking place in Southern Niagara, ON, Canada. The NLHS is a follow‐up study that builds on three baseline studies that took place across the Niagara Region from 2007 to 2012. Each baseline study varied in its collection of demographic, psychosocial, lifestyle, and biological measures; however, all three studies collected data on various CV health parameters in a subset of their respective study populations (Wade et al., 2019). At baseline, CV data were obtained on 564 individuals who ranged in age from 8 to18 years. It is these individuals (now aged 19–25 years) who are currently being re‐recruited for follow‐up CV assessment. However, due to the perceived intrusiveness in the acquisition of the femoral PP in children and adolescents, cfPWV was not measured at baseline, thereby precluding the examination of change in cfPWV longitudinally (Wade et al., 2019). To date, the NLHS has re‐recruited 248 individuals for follow‐up assessment, with complete cfPWV and MMP‐3 data being available for 206 individuals.

As per the NLHS testing protocol (Wade et al., 2019), all participants were instructed to avoid vigorous physical activity as well as alcohol and caffeine consumption for at least 12 h prior to their laboratory visit. Participants were also asked to fast for at least 4 h prior to testing. The NLHS protocol was approved by the Brock University Biological Research Ethics Board and all participants gave written informed consent.

### 2.2 Blood pressure, heart rate, and anthropometric measures

Testing was performed in a quiet, dimly lit, and temperature‐controlled room. After a period of 15 min of quiet rest in a seated position, six heart rate (HR) and BP measurements were taken at 1‐min intervals using an automated oscillometric device (BpTRU Vital Signs Monitor, BPM‐300, VSM, MedTech Devices). Of the six measurements, only the final three values were used to calculate seated mean systolic and diastolic BP (SBP and DBP, respectively) as well as seated mean arterial pressure [MAP = DBP + 1/3(SBP − DBP)].

Standing height (cm) was measured using a stadiometer (STAT‐7X, Ellard Instrumentations) and body mass (kg) was measured using a digital scale (BWB‐800S, Tanito). Body mass index (BMI) (kg/m^2^) was calculated as body mass (kg) divided by height squared (m^2^). BMI was categorized as normal weight (<25 kg/m^2^), overweight (25–29.9 kg/m^2^), and obese (>30 kg/m^2^). Information on smoking status was collected by asking the question: “*Do you currently smoke cigarettes daily*, *occasionally or not at all?”* Smoking status was then categorized into three groups: regular smokers, occasional smokers, and non‐smokers. Regular smokers were defined as those who smoked at least one cigarette per day, occasional smokers were defined as those who had smoked less than one cigarette per day, and non‐smokers were defined as those who did not smoke cigarettes.

### 2.3 Assessment of central arterial stiffness

Measurement of cfPWV was used to assess central arterial stiffness and was defined as the speed of the pulse from the left common carotid artery (CCA) to the left femoral artery. This method is based on the principal that pressure waves generated during left ventricular ejection will travel with a faster velocity through stiffer arteries compared to more elastic arteries. To obtain cfPWV, local PP waveforms were collected at the left CCA and femoral arterial sites using a handheld applanation tonometer (Millar Instruments).

Concurrent with PP waveform acquisition, instantaneous beat‐by‐beat BP and HR measures were recorded using a finger BP cuff placed on the left middle digit (Nexfin, BMEYE; NIBP Nano, ADInstruments) and a standard single‐lead electrocardiogram (ECG), respectively. One‐minute averages of beat‐by‐beat BP and HR recordings were calculated to obtain mean instantaneous BP and HR. cfPWV was calculated using the formula: *cfPWV* = *∆d*/*∆t*, where *∆d* was the sum of the distance from the suprasternal notch to the umbilicus and the umbilicus to the femoral artery minus the distance from the suprasternal notch to the CCA in meters (m), and *∆t* was the pulse transit time (PTT) to the femoral artery minus the PTT to the CCA in seconds (s). Distance measurements were obtained using an inelastic tape and the PTT was determined as the foot of the pressure wave corresponding to the foot of the systolic upstroke of the CCA and femoral arterial signals relative to the R‐wave of the ECG. Arterial signals were passed through a band‐pass filter of 5–30 Hz and the foot of the waveform was identified as the minimum value of the filtered signal (Currie et al., 2010; Munakata, 2003). Three consecutive sequences of at least 15 PP waveforms were obtained at both the CCA and femoral arterial sites using the handheld tonometer, and 15 of the most well‐defined and consistent waveforms were averaged in the calculation of the PTT. Values for cfPWV are expressed in m/s.

### 2.4 Assessment of serum MMP‐3 and serum C‐reactive protein

As per the NLHS testing protocol, 15 ml of venous blood was drawn from each participant by a licensed nurse. Serum was separated from 5 ml of blood by centrifugation (1500*g* at 4℃ for 10 min) and stored at −80℃. A commercially available enzyme‐linked immunosorbent assay (ELISA) (Bio‐Rad Laboratories) was used for the quantification of total serum MMP‐3 concentrations. Total serum C‐reactive protein (CRP) concentrations were quantified using a commercially available human CRP ELISA kit (R&D Systems). Per the manufacturer, the mean intra‐ and inter‐assay coefficients of variation of the MMP‐3 assay are 3.7% and 8.8%, respectively, and the lowest detectable limit of serum MMP‐3 is 0.029 ng/ml, while the mean intra‐ and inter‐assay coefficients of variation for the CRP assay are 5.5% and 6.5%, respectively, and the lowest detectable limit of serum CRP is 0.022 ng/ml.

### 2.5 Statistical analysis

Continuous variables are presented as the mean ± the standard deviation (SD) and categorical variables are presented as proportions and relative frequencies (%). Student's *t* tests were used to examine differences in mean serum MMP‐3 concentrations and mean cfPWV between sexes. Analyses of variance followed by Tukey's post hoc tests were used to examine differences in mean serum MMP‐3 concentrations and mean cfPWV between smoking status groups and by BMI classification. Pearson correlation coefficients were calculated to examine bivariate associations between the dependent and independent variables, and multiple linear regression analyses were used to examine the association between cfPWV (dependent variable) and serum MMP‐3 (independent variable) after adjustment for several covariates, including age, sex, smoking status, BMI, instantaneous MAP and HR, and serum CRP (Model 1). The interaction terms MMP‐3 by smoking status (Model 2), MMP‐3 by BMI (Model 3), MMP‐3 by sex (Model 4), and MMP‐3 by instantaneous MAP (Model 5) were added in subsequent regression models in order to determine the potential interaction effects between these factors in predicting cfPWV. The sample size required to detect a small‐to‐medium effect size (Cohen's *f* ^2^ = 0.15) at an *α*‐level of 0.05 and a power (1 − *β*) of 0.90 was 152 individuals (Faul et al., 2009). *p*‐values <0.05 were considered statistically significant. All statistical analyses were performed using SAS 9.4 (SAS Institute).

## 3 RESULTS

### 3.1 Participant characteristics

The characteristics of the study population can be observed in Table 1. A total of 206 participants were included in the analysis (*n* = 109 females). Mean cfPWV of the sample was 5.9 ± 1.0 m/s, which is considered normal according to The Reference Values for Arterial Stiffness’ Collaboration (2010). Mean serum MMP‐3 concentration of the sample was 4.5 ± 2.7 ng/ml, which is consistent with other studies that have published serum MMP‐3 concentrations in healthy populations (Preston et al., 2002; Thrailkill et al., 2005).

**TABLE 1:** Characteristics of the study population (*n* = 206)

Variable	Parameter	Range	IQR
Age (years)	22.6 ± 1.5	19.1–25.6	2.5
Sex [*n* (%)]^a^			
Males	97 (47.1)	—	—
Females	109 (52.9)	—	—
Smoking Status [*n* (%)]^a^			
Daily smokers	19 (9.2)	—	—
Occasional smokers	16 (7.8)	—	—
Non‐smokers	171 (83.0)	—	—
Standing height (m)	1.7 ± 0.1	1.5–1.9	0.1
Body mass (kg)	75.3 ± 19.0	43.4–135.9	23.8
BMI (kg/m^2^)	25.7 ± 6.1	16.4–42.4	6.9
BMI classification [*n* (%)]^a^			
Normal weight	117 (56.8)	—	—
Overweight	53 (25.7)	—	—
Obese	36 (17.5)	—	—
MAP (mmHg)	83.1 ± 8.9	56.9–109.6	11.3
SBP (mmHg)	115.9 ± 11.4	92.0–152.7	14.6
DBP (mmHg)	66.8 ± 9.3	38.7–88.0	12.3
HR (bpm)	65.5 ± 10.3	38.9–108.1	12.5
cfPWV (m/s)	5.9 ± 1.0	3.7–10.2	1.1
Serum CRP (ng/ml)	3.1 ± 4.9	0.1–25.0	2.9
Serum MMP‐3 (ng/ml)	4.5 ± 2.7	0.4–15.1	2.9

Abbreviations: BMI, body mass index; cfPWV, carotid‐femoral pulse wave velocity; CRP, C‐reactive protein; DBP, instantaneous diastolic blood pressure; HR, instantaneous heart rate; IQR, the interquartile range; MAP, instantaneous mean arterial pressure; MMP‐3, matrix metalloproteinase 3; SBP, instantaneous systolic blood pressure.

^a^ Categorical variables are presented as proportions and relative frequencies, and continuous variables are presented as mean values ±SD.

### 3.2 Bivariate associations of serum MMP3 and cfPWV

**FIGURE 1:**
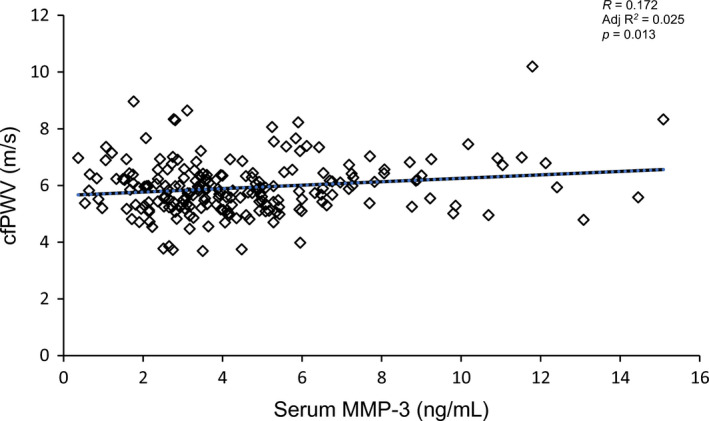
Association between cfPWV (m/s) and serum MMP‐3 concentration (ng/mL) (unadjusted)

In bivariate analysis, serum MMP‐3 concentrations were significantly and positively associated with cfPWV (*r* = 0.172 *p* = 0.013) (Figure 1). Additionally, serum MMP‐3 concentrations were associated with instantaneous MAP (*r* = 0.216, *p* = 0.002), SBP (*r* = 0.193, *p* = 0.006), and DBP (*r* = 0.191, *p* = 0.007). Serum MMP‐3 concentrations were not associated with age, BMI, HR, or serum CRP concentrations (all *p* > 0.05). Between‐groups analysis revealed that mean serum MMP‐3 concentrations significantly differed by sex, such that males had significantly higher serum MMP‐3 concentrations compared to females (5.71 ± 2.98 vs. 3.46 ± 1.90 ng/ml, respectively, *p* < 0.001). Serum MMP‐3 concentrations did not differ by smoking status nor BMI classification (both *p* > 0.05). As expected, cfPWV was significantly and positively associated with instantaneous MAP (*r* = 0.238, *p* = 0.001), SBP (*r* = 0.294, *p* < 0.001), and DBP (*r* = 0.162, *p* = 0.021). Additionally, cfPWV was associated with age (*r* = 0.218, *p* = 0.002), BMI (*r* = 0.285, *p* < 0.001), HR (*r* = 0.236, *p* = 0.001), serum CRP concentrations (*r* = 0.229, *p* = 0.001), and regular smoking (*r* = 0.245, *p* < 0.001). Between‐groups analysis revealed that mean cfPWV significantly differed by smoking status, such that regular smokers had a significantly faster cfPWV compared to non‐smokers (6.65 ± 1.29 vs. 5.81 ± 0.85 m/s, respectively; *p* = 0.012), but not occasional smokers (6.65 ± 1.29 vs. 6.21 ± 1.15 m/s, respectively; *p* > 0.05). There was no significant difference in mean cfPWV between occasional smokers and non‐smokers (*p* > 0.05). Mean cfPWV also significantly differed by BMI classification, such that those classified as obese had a significantly faster cfPWV compared to both overweight and normal weight individuals (6.46 ± 1.23 vs. 5.85 ± 0.67 and 5.79 ± 0.92 m/s, respectively; *p* < 0.05). There was no significant difference in mean cfPWV between overweight and normal weight individuals (*p* > 0.05). cfPWV did not significantly differ by sex (*p* > 0.05).

### 3.3 Association between serum MMP‐3 and cfPWV

Multiple linear regression analyses were used to examine the association of serum MMP‐3 concentration on cfPWV after adjustment for several covariates (i.e., age, sex, smoking status, BMI, instantaneous MAP and HR, and CRP) (Model 1). Serum concentrations of CRP were also adjusted for, as subclinical inflammation has been associated with arterial stiffness (Schumacher et al., 2009; Yasmin et al., 2004) and may also affect serum MMP concentrations (Vlachopoulos et al., 2007). In this model, serum MMP‐3 remained a significant and positive predictor of cfPWV (*β* = 0.187, *p* = 0.007), independent of age, sex, smoking status, BMI, instantaneous MAP and HR, and serum CRP (Table 2; Model 1). Age, regular smoking, BMI, and HR were also significant predictors of cfPWV in this model (all *p* < 0.05) (Table 2; Model 1). Together, serum MMP‐3, age, regular smoking, BMI, and HR were able to predict 22.9% of the variation in cfPWV (Adj. *R* ^2^ = 0.229, *p* < 0.001). In a model that included SBP instead of MAP as a predictor of cfPWV, serum MMP‐3 remained a significant predictor of cfPWV, independent of age, sex, smoking status, BMI, HR, and serum CRP (*β* = 0.193, *p* = 0.005; data not shown).

**TABLE 2:** Linear regression main effect and interaction models examining cfPWV on serum MMP‐3 and covariates (*n* = 206)

Model	Variable	*B* (SE)	*β*	95% CI		*p*
				LL	UL	
Model 1^c^	Age (years)	0.142 (0.040)	0.225	0.063	0.222	0.001^●^
	Sex^a^	−0.138 (0.145)	−0.072	−0.423	0.148	0.344
	Reg. Smoke^b^	0.700 (0.218)	0.213	0.270	1.131	0.002^●^
	Occ. Smoke^b^	0.321 (0.224)	0.090	−0.121	0.763	0.154
	BMI	0.025 (0.012)	0.160	0.002	0.048	0.034^●^
	MAP	0.015 (0.007)	0.137	−0.001	0.029	0.052
	HR	0.017 (0.006)	0.185	0.005	0.029	0.004^●^
	Serum CRP	0.012 (0.015)	0.062	−0.017	0.041	0.421
	Serum MMP‐3	0.066 (0.024)	0.187	0.018	0.114	0.007^●^
Model 2^c^	Age (years)	0.138 (0.040)	0.217	0.059	0.216	0.001^●^
	Sex^a^	−0.090 (0.144)	−0.047	−0.375	0.194	0.533
	Reg. Smoke^b^	−0.153 (0.427)	−0.047	−0.994	0.688	0.720
	Occ. Smoke^b^	−0.305 (0.489)	−0.086	−1.269	0.659	0.533
	BMI	0.021 (0.012)	0.136	−0.002	0.044	0.069
	MAP	0.013 (0.007)	0.117	−0.002	0.027	0.093
	HR	0.018 (0.006)	0.198	0.007	0.030	0.002^●^
	Serum CRP	0.021 (0.015)	0.109	−0.009	0.051	0.164
	Serum MMP‐3	0.038 (0.026)	0.109	−0.013	0.090	0.144
	MMP‐3*Occ. Smoke	0.119 (0.079)	0.212	−0.036	0.274	0.131
	MMP‐3*Reg. Smoke	0.176 (0.076)	0.291	0.026	0.327	0.022^●^
Model 3^c^	Age (years)	0.140 (0.040)	0.221	0.062	0.219	0.001^●^
	Sex^a^	−0.069 (0.146)	−0.036	−0.357	0.218	0.634
	Reg. Smoke^b^	0.628 (0.217)	0.191	0.199	1.057	0.004^●^
	Occ. Smoke^b^	0.298 (0.222)	0.084	−0.139	0.735	0.180
	BMI	0.003 (0.015)	0.017	−0.026	0.031	0.856
	MAP	0.015 (0.007)	0.136	0.001	0.029	0.049^●^
	HR	0.015 (0.006)	0.163	0.003	0.027	0.011^●^
	Serum CRP	0.021 (0.015)	0.106	−0.009	0.050	0.173
	Serum MMP‐3	0.045 (0.025)	0.128	−0.005	0.095	0.077
	MMP‐3*BMI	0.098 (0.039)	0.210	0.021	0.175	0.013^●^

Abbreviations: “^●^”, significant *p*‐values (*p* < 0.05); *B*, the unstandardized regression coefficient; BMI, body mass index; cfPWV, carotid‐femoral pulse wave velocity; CRP, C‐reactive protein; HR, instantaneous heart rate; LL, is the lower limit of the 95% confidence interval (95% CI); MAP, instantaneous mean arterial pressure; MMP‐3, matrix metalloproteinase 3; Occ. Smoke, occasional smoking; Reg. Smoke, regular smoking; SE, standard error; UL, upper limit; *β*, standardized regression coefficient.

^a^ Reference group for sex is male.

^b^ Reference group for smoking status is non‐smokers.

^c^ Adjusted *R* ^2^ of Model 1 = 0.239, *p* < 0.001; adjusted *R* ^2^ of Model 2 = 0.258, *p* < 0.001; adjusted *R* ^2^ of Model 3 = 0.246, *p* < 0.001.

### 3.4 Interactions of serum MMP‐3 with smoking, BMI, sex, and MAP in predicting cfPWV

**FIGURE 2:**
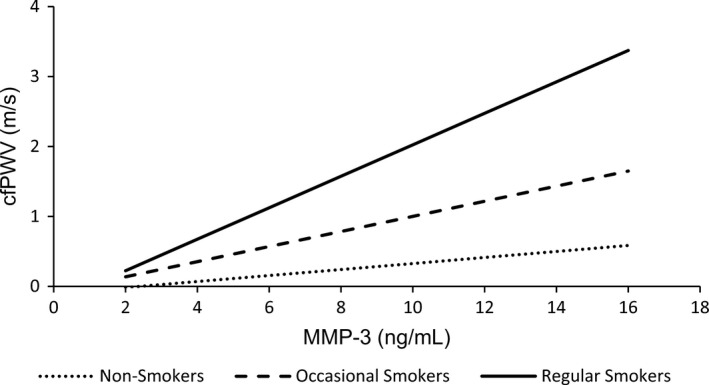
Interaction between smoking status and serum MMP‐3 in predicting cfPWV, adjusted for age, sex, BMI, instantaneous MAP and HR, and serum CRP (Model 2)

**FIGURE 3:**
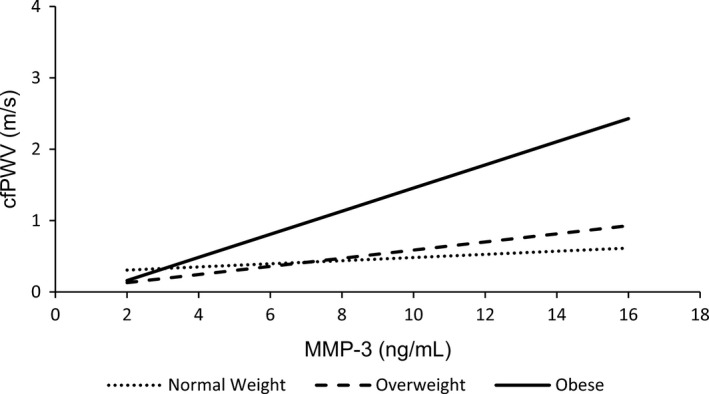
Interaction between BMI classification and serum MMP‐3 in predicting cfPWV, adjusted for age, sex, smoking status, instantaneous MAP and HR, and serum CRP (Model 3)

Interactions between serum MMP‐3 and smoking status (Model 2), BMI (Model 3), sex (Model 4), and MAP (Model 5) were examined in subsequent regression models. There was no significant interaction between serum MMP‐3 and sex (*p* > 0.05; Model 4; data not shown) nor serum MMP‐3 and MAP (*p* > 0.05; Model 5; data not shown) in predicting cfPWV. Model 2 revealed a significant interaction between serum MMP‐3 and regular smoking (*β* = 0.291, *p* = 0.022) in predicting cfPWV, but not occasional smoking (*β* = 0.212, *p* = 0.131) (Table 2). The smoking by serum MMP‐3 interaction can be observed in Figure 2. In Model 3 there was a significant interaction between serum MMP‐3 and BMI in predicting cfPWV (*β* = 0.210, *p* = 0.013) (Table 2). The BMI by serum MMP‐3 interaction can be observed in Figure 3.

## 4 DISCUSSION

The current study revealed a significant and positive association between serum MMP‐3 concentration and central arterial stiffness as measured by cfPWV in a sample of healthy young adults. This association remained significant after adjustment for several covariates, including age, sex, smoking status, BMI, instantaneous MAP and HR, and serum CRP. This study also revealed a significant interaction between serum MMP‐3 and smoking, and serum MMP‐3 and BMI in predicting cfPWV.

Stiffening of the large arteries is associated with a widening of the PP and alterations in shear stress that may ultimately contribute to an increased CVD risk (Nichols & O’Rourke, 2005). As previously described, the ECM of large arteries contains a number of structural proteins, with elastin and collagen being the two main components (Nichols & O’Rourke, 2005; Zieman et al., 2005). Elastin composes the main elastic element of the arterial wall and provides the large arteries with a significant buffering capacity (Nichols & O’Rourke, 2005; Yasmin et al., 2005). Elastin fibers have been shown to have a very low turnover rate in vivo, allowing for the accumulation of structural changes caused by fragmentation and MMP degradation (Kohn et al., 2015). As these structural changes accumulate, elastin loses its functionality and shifts load bearing onto the much stiffer collagen fibers, thereby directly contributing to arterial stiffness (Kohn et al., 2015). There is also evidence that MMPs play a role in elastin calcification within the arterial wall, which may further contribute to arterial stiffening (Bailey et al., 2004; Basalyga et al., 2004; Cui et al., 2000). MMP‐3, in particular, may contribute to the progression of central arterial stiffness by degrading elastin within the arterial wall ECM, participating in calcium mineralization, and by activating downstream MMPs, such as MMP‐1 and MMP‐9, which further exacerbate vascular remodeling and arterial stiffness (Cui et al., 2000; Medley et al., 2003).

Previous studies examining the association between MMP‐3 and arterial health have largely focused on a two‐allele promoter polymorphism involving a run of either five or six adenosines (5A/6A) within the *MMP3* gene (Kingwell et al., 2001; Medley et al., 2003). Functional studies of the 5A/6A polymorphism have indicated that the 5A‐allele has a higher promoter activity than the 6A‐allele in both cultured fibroblasts and VSMCs in vitro (Ye et al., 1996)—a finding bolstered by gene expression studies in skin biopsies that have also found higher MMP‐3 activity in 5A‐allele carriers (Medley et al., 2003). Accordingly, it has been postulated that the 5A‐allele is associated with higher levels of circulating MMP‐3 compared to the 6A‐allele, and indeed some studies, but not all (Du et al., 2020; Gnasso et al., 2000; Samnegard et al., 2005), have found this hypothesis to hold true (El‐Aziz & Mohamed, 2016; Medley et al., 2003). Furthermore, it has been speculated that the 5A‐allele contributes to a vascular phenotype characterized by increased ECM degradation, whereas the 6A‐allele contributes to a vascular phenotype characterized by increased ECM deposition (Ye, 2006), although genetic association studies have also reported somewhat conflicting findings (Beton et al., 2016; Beyzade et al., 2003; Nojiri et al., 2003; Samnegard et al., 2005; Yamada et al., 2002). Thus, it may be important to consider serum MMP‐3 concentrations when examining the role of MMP‐3 in tissue remodeling and maintenance in the context of CV health and disease.

To date, few studies have examined the association between MMP‐3 and measures of arterial stiffness (Medley et al., 2003; Peeters et al., 2017; Rajzer et al., 2017), and none have examined the effects of serum MMP‐3 concentration on central arterial stiffness in a population of healthy adults, specifically. Of note, Medley et al. (2003) found that in a sample of older adults (aged >60 years; *n* = 77) at low CVD risk, aortic stiffness measured by aortic impedance was greater in 5A‐ and 6A‐allele homozygotes compared to heterozygotes; a finding that was not replicated in younger individuals (aged 30–60 years; *n* = 126) (Medley et al., 2003). The authors also found that *MMP3* gene expression was significantly different between genotypes, such that the 6A/6A genotype was associated with a 2‐fold lower expression and the 5A/5A genotype was associated with a 4‐fold higher expression compared to the 5A/6A genotype (Medley et al., 2003). Although *MMP3* gene expression does not necessarily correspond to MMP‐3 activity, the authors suggested that either high or low MMP‐3 activity may be suboptimal with regard to central arterial stiffness (Medley et al., 2003).

Several studies have provided evidence that circulating concentrations of various MMPs can influence arterial stiffness (McNulty et al., 2006; Peeters et al., 2017; Rajzer et al., 2017; Yasmin et al., 2005). For example, McNulty et al. (2006) found evidence that plasma MMP‐1 (i.e., collagenase‐1) concentrations were positively associated with cfPWV in both hypertensive (*n* = 32, mean age 49 ± 2 years) and normotensive (*n* = 14, mean age 44 ± 3 years) individuals, independent of age and MAP. Similarly, Yasmin et al. (2005) found that serum MMP‐2 and serum MMP‐9 (i.e., gelatinases A and B, respectively) concentrations were positively associated with cfPWV in a hypertensive population (*n* = 116, mean age 68 ± 8 years), independent of age and MAP. Additionally, these authors found serum MMP‐9 to be positively associated with cfPWV in a sample of normotensive adults (*n* = 447, mean age 48 ± 18 years), independent of age, sex, MAP, and serum CRP concentrations (Yasmin et al., 2005). Collectively, these studies demonstrate an association of circulating MMPs with central arterial stiffness.

Serum MMP‐3 concentrations have also been linked to central arterial stiffness. Indeed, Razjer et al. (2017) found that serum MMP‐3 concentrations were positively associated with cfPWV in hypertensive individuals (*n* = 95, mean age 53 ± 13 years), but not normotensive individuals (*n* = 31, mean age 53 ± 13 years), independent of age, sex, BP, BMI, serum MMP‐2, −9 and proMMP‐1, total cholesterol, and serum LDL (Rajzer et al., 2017). Additionally, Peeters et al. (2017) found a positive association between serum MMP‐3 concentration and cfPWV in a population of type I diabetics (*n* = 614, mean age 54 ± 9 years), independent of age, sex, MAP, BMI, smoking status, total cholesterol, use of antihypertensive medication, and other diabetic and vascular covariates (Peeters et al., 2017). In the current study, it was found that serum MMP‐3 was significantly and positively associated with cfPWV in a population of healthy young adults, independent of age, sex, smoking status, BMI, instantaneous MAP and HR, and serum CRP, a finding that contradicted that of Razjer et al. who had found no association between MMP‐3 and cfPWV in a healthy population (Rajzer et al., 2017). Potential reasons for these contrasting findings may be related to the smaller sample used in the study by Razjer et al. (*n* = 31), differences in study population demographics, or differences in pre‐analytical sampling conditions (i.e., sampling of serum vs. plasma for the assessment of circulating MMP‐3 concentrations) (Jung, 2005; Mannello, 2008). Indeed, there is evidence that blood sampling and handling can influence the concentrations of circulating MMPs, such that some MMPs exist in significantly higher concentrations in serum compared to plasma (Jung, 2005; Jung et al., 2005; Mannello, 2008). For example, MMP‐9 has been shown to be significantly higher in serum compared to plasma on several occasions (Jonsson et al., 2016; Jung et al., 2006)—a finding attributed to MMP‐9 secretion by platelets and leukocytes during coagulation and fibrinolysis in serum processing (Jonsson et al., 2016; Mannello, 2008). However, it may be inappropriate to generalize the findings related to MMP‐9 to all MMP measurements (Thrailkill et al., 2006). It has been shown that for MMP‐3 in particular, serum and plasma concentrations are highly correlated (Thrailkill et al., 2006) and sampling method does not play a significant role in determining circulating MMP‐3 levels (Mannello, 2008; Thrailkill et al., 2006). Thus, the analysis of serum MMP‐3 likely provides similar information to that of plasma MMP‐3 (Mannello, 2008; Thrailkill et al., 2006).

Another key finding of the current study was the significant interaction effect between smoking and serum MMP‐3 in predicting cfPWV. Previous studies that have examined the interaction between cigarette smoking and MMP‐3 in the context of cardiovascular health have focused on the interaction between smoking and the *MMP3* 5A/6A promoter polymorphism in coronary heart disease (CHD) risk (Humphries et al., 2002; Liu et al., 2003). Humphries et al. (2002) found a significant interaction effect between smoking and the 5A/5A genotype in evaluating the risk of CHD‐related events, such as acute myocardial infarction, in healthy middle‐aged men (*n* = 473). The authors also revealed that smokers with the 5A/5A genotype had a 3.6‐fold higher risk of acute myocardial infarction, when compared to non‐smokers with the 5A/5A genotype, even after adjustment for BP, BMI and cholesterol, triglyceride, and fibrinogen levels (Humphries et al., 2002). Similarly, Liu et al. (2003) found that smokers carrying the 5A‐allele had a 10‐fold greater risk of acute myocardial infarction compared to non‐smoking non‐carriers (Liu et al., 2003).

In contrast to these studies, the current study examined the interaction between smoking and serum MMP‐3 on central arterial stiffness. It was found that smoking modulated the association between serum MMP‐3 and cfPWV, such that the association was the strongest in regular smokers. Moreover, although there was no significant interaction between occasional smoking and serum MMP‐3 in predicting cfPWV, the current study provided preliminary evidence of a modulatory effect of smoking intensity on the interaction between smoking and serum MMP‐3 in predicting cfPWV (Figure 2). Future studies should utilize more robust measures of smoking intensity (e.g., packs per day or pack‐years) to explore the effects of smoking on the association between serum MMP‐3 and central arterial stiffness. Interestingly, there was no evidence in the current study to suggest that smokers had higher concentrations of serum MMP‐3 compared to either occasional or non‐smokers, although this finding may be partly attributable to the low number of smokers included in the study (*n* = 19).

Smoking‐induced inflammation is a plausible mechanism by which smoking influences the association between MMP‐3 and arterial stiffness (Doonan et al., 2010; Humphries et al., 2002; Perlstein & Lee, 2006). It is possible that smoking‐induced vascular injury upregulates MMP‐3 within immune cells (e.g., monocytes/macrophages) and cells of the vasculature (e.g., VECs and VSMCs) in order to promote tissue repair and compensatory remodeling. Smoking triggers an immune response to vascular injury, which has been associated with increased levels of circulating inflammatory markers, such as CRP, IL‐6, TNF*α*, and IL‐1*β* (Barbieri et al., 2011; Levitzky et al., 2008), and an increased leukocyte count (Pedersen et al., 2019). Interactions between macrophages with cells of the vasculature, specifically VECs and VSMCs, may play a critical role in smoking‐induced MMP‐3 expression in the context of arterial stiffening (Bond et al., 2001; Chase et al., 2002; Lee et al., 1995). Indeed, the upregulation of MMP‐3 in both macrophages (Chase et al., 2002) and VSMCs (Bond et al., 2001) is dependent on the activation of NF‐κB, and the activation of NF‐κB is directly associated with cigarette smoking (Wang et al., 2015). Downstream, activation of NF‐κB is also associated with the upregulation of IL‐1*β* in macrophages (Wang et al., 2015; Xu et al., 2011), which has also been shown to stimulate MMP‐3 production in VSMCs (Lee et al., 1995). Components of cigarette smoke (e.g., nicotine, cotinine, and smoke particulate matter) have also been shown to directly upregulate the expression of MMP‐3 in both VSMCs (Carty et al., 1996) and VECs (Bishop et al., 2012).

In addition to the interaction between cigarette smoking and serum MMP‐3 in predicting cfPWV, a significant interaction between serum MMP‐3 and BMI was also noted. Indeed, it was found that as BMI increased, the association between serum MMP‐3 and cfPWV became stronger. Interestingly, this interaction appeared to be driven by those who were considered obese (Figure 3). Increasing adiposity and obesity are strongly associated with systemic low‐grade inflammation pertinent to endothelial dysfunction, vascular remodeling, and arterial stiffening (Brunner et al., 2015; García‐Prieto et al., 2019). Indeed, circulating levels of several inflammatory cytokines (i.e., CRP, IL‐1*β*, IL‐6, IL‐8, MCP‐1, and TNF*α*) have been shown to be increased in obesity (Lacasa et al., 2007; O’Hara et al., 2012). It has been suggested that some of these cytokines, specifically IL‐1*β*, IL‐6, and TNF*α*, largely originate from the stromal vascular fraction, which includes infiltrated macrophages, among other cell types (Curat et al., 2006; Lacasa et al., 2007). Macrophages are an important contributor to obesity‐associated inflammation (Curat et al., 2006; O’Hara et al., 2012), and the cross‐talk between macrophages and adipocytes/preadipocytes may play a key role in the progression of obesity‐associated central arterial stiffness, particularly in the context of MMP‐3‐associated ECM remodeling (O’Hara et al., 2009, 2012).

Previous studies, but not all (Maquoi et al., 2003; Wu et al., 2017), that have examined the association between MMP‐3 and obesity have found MMP‐3 to be upregulated in mice with nutritionally induced (Maquoi et al., 2002) and genetic obesity (i.e., *ob*/*ob* and *db*/*db* mice) (Chavey et al., 2003; Unoki et al., 2006). This upregulation in MMP‐3 may be partly attributable to the milieu of inflammatory factors secreted by macrophages in obesity (Bing, 2015; O’Hara et al., 2009, 2012). For example, in addition to stimulating MMP‐3 expression in VSMCs, IL‐1*β* has been shown to induce MMP‐3 expression in preadipocytes and adipocytes (Gao & Bing, 2011; O’Hara et al., 2009, 2012). Downstream, IL‐1*β* acts in an autocrine/paracrine manner to stimulate IL‐6 expression in adipocytes and circulating leukocytes (Flower et al., 2003; Lacasa et al., 2007), whereas MMP‐3 stimulates TNF*α* expression in preadipocytes (Lacasa et al., 2007; O’Hara et al., 2009; Unoki et al., 2006). IL‐1*β*, IL‐6, and TNF*α* have been associated with cfPWV in different populations (Mahmud & Feely, 2005; Tuttolomondo et al., 2015), suggesting that inflammation associated with increasing adiposity could partly explain the stronger association between serum MMP‐3 concentrations and cfPWV in individuals with larger BMIs.

### 4.1 Potential limitations

In the current study, serum MMP‐3 concentration rather than activity was measured. However, MMP‐3 activity is inhibited by several TIMPs, including TIMPs‐1, −2, −3, and −4, which provide a regulatory mechanism by which excessive ECM degradation is prevented (Nagase, 2013). Imbalance in the MMP‐TIMP system due to the upregulation of MMP‐3 may lead to ECM degradation within the arterial wall and thereby contribute to central arterial stiffness (Kohn et al., 2015); however, the downregulation of TIMP activity may produce a similar result (Peeters et al., 2017). Although serum TIMP concentrations were not measured in the current study, previous studies have not found TIMP levels to be significantly associated with cfPWV (Peeters et al., 2017; Yasmin et al., 2005), suggesting that increased MMP concentrations, rather than changes in TIMP concentrations, are more likely contributors to arterial stiffness (Yasmin et al., 2005). Nevertheless, measurement of serum TIMPs may provide further insights into the observed association between serum MMP‐3 and central arterial stiffness, and future studies should aim to include measurements of serum TIMP concentrations. The *MMP3* 5A/6A promoter polymorphism likely also plays a critical role in the expression and activity of MMP‐3 (Medley et al., 2003). Although the aim of the current study was to examine the association between serum MMP‐3 concentrations and central arterial stiffness, future studies should consider *MMP3* genotype as a potential covariate in this association, as differences in protein expression between the various genotypes have been observed (Medley et al., 2003; Samnegard et al., 2005). Regardless, the results of the current study largely corroborate the findings of previous studies, that have revealed significant positive associations between circulating MMP concentrations and central arterial stiffness (Peeters et al., 2017; Yasmin et al., 2005). Finally, due to the cross‐sectional nature of the current study, it is difficult to infer a causal relationship between serum MMP‐3 concentrations and central arterial stiffness. Further experimental models are needed to determine the exact mechanisms by which MMPs contribute to the remodeling of the arterial wall ECM and how this remodeling contributes to the progression of central arterial stiffness. Moreover, future studies should examine the role of macrophages and other inflammatory mediators (e.g., cytokines/chemokines, growth factors, and MMPs) in tissue remodeling and central arterial stiffness in the context of smoking‐induced and/or obesity‐associated inflammation.

## 5 CONCLUSION

The current study showed for the first time that serum MMP‐3 concentrations are significantly and positively associated with central arterial stiffness measured by cfPWV in a population of healthy young adults. Moreover, the current study revealed a significant interaction effect between serum MMP‐3 and smoking, and serum MMP‐3 and BMI in predicting cfPWV. This suggests that the association between MMP‐3 and cfPWV may be influenced by factors associated with a pro‐inflammatory state. Although speculative, such factors may, in part, drive MMP‐mediated arterial stiffening.

## CONFLICT OF INTEREST

The authors declare no competing interests associated with this manuscript.

## AUTHOR CONTRIBUTIONS

Data were collected and analyzed at Brock University. TJW and DDO are principal investigators for the NLHS on which this analysis was based. Anthropometric and cardiovascular data were collected and analyzed by KSD and NJI. ELISAs were performed by JM and NJI under the direction of AJM. NJI, AJM, and DDO were responsible for the conceptualization and data analysis of the current work, with NJI drafting the original manuscript. All authors were involved in interpretation of the data and revising of the manuscript. All authors approved the final version of the manuscript and agree to be accountable for all aspects of the work.
